# Pro- and Antioxidant Functions of the Peroxisome-Mitochondria Connection and Its Impact on Aging and Disease

**DOI:** 10.1155/2017/9860841

**Published:** 2017-07-24

**Authors:** Amparo Pascual-Ahuir, Sara Manzanares-Estreder, Markus Proft

**Affiliations:** ^1^Department of Biotechnology, Instituto de Biología Molecular y Celular de Plantas, Universitat Politècnica de València-CSIC, Ingeniero Fausto Elio s/n, 46022 Valencia, Spain; ^2^Department of Molecular and Cellular Pathology and Therapy, Instituto de Biomedicina de Valencia IBV-CSIC, Jaime Roig 11, 46010 Valencia, Spain

## Abstract

Peroxisomes and mitochondria are the main intracellular sources for reactive oxygen species. At the same time, both organelles are critical for the maintenance of a healthy redox balance in the cell. Consequently, failure in the function of both organelles is causally linked to oxidative stress and accelerated aging. However, it has become clear that peroxisomes and mitochondria are much more intimately connected both physiologically and structurally. Both organelles share common fission components to dynamically respond to environmental cues, and the autophagic turnover of both peroxisomes and mitochondria is decisive for cellular homeostasis. Moreover, peroxisomes can physically associate with mitochondria via specific protein complexes. Therefore, the structural and functional connection of both organelles is a critical and dynamic feature in the regulation of oxidative metabolism, whose dynamic nature will be revealed in the future. In this review, we will focus on fundamental aspects of the peroxisome-mitochondria interplay derived from simple models such as yeast and move onto discussing the impact of an impaired peroxisomal and mitochondrial homeostasis on ROS production, aging, and disease in humans.

## 1. Introduction

Oxidative stress is causally linked to accelerated aging and aging-related diseases [1]. In eukaryotic cells, mitochondria and peroxisomes are the main ROS contributors [2, 3]. At the same time, both organelles are equipped with their own ROS scavenging repertoire of enzymes. Mitochondria and peroxisomes are metabolically linked because they share common pathways such as the fatty acid *β*-oxidation. However, both organelles are much more intimately regulated with respect to ROS homeostasis and the clearance of dysfunctional organelles. Here, we will focus on our current knowledge of mitochondrial and peroxisomal pro- and antioxidant functions, their coordinated turnover by autophagic pathways, and their functional and physical interactions. Investigation in the yeast model is contributing fundamental insights into the mechanisms of quality control of peroxisomes and mitochondria. Therefore, we will compare the dynamics of both organelles and its impact on cell survival and health in yeast and mammalian cells throughout the review.

## 2. Pro- and Antioxidant Functions of Peroxisomes

Peroxisomes are versatile organelles found in most eukaryotic cells. The name peroxisome has been originally introduced for a cellular organelle which contains at least one H_2_O_2_-producing oxidase and the H_2_O_2_-detoxifying enzyme catalase [4]. Today, we know that peroxisomal function goes far beyond the metabolism of reactive oxygen species. Peroxisomes contain more than 50 different enzymatic activities, which shape important metabolic and anabolic functions, including fatty acid oxidation and lipid biosynthesis. As they share an oxidative metabolism together with mitochondria, they are key organelles in the cellular homeostasis of ROS [5, 6]. Although the variability of the metabolic activities contained by peroxisomes of different organisms or tissues is considerable, their universal functions are the oxidative metabolism of fatty acids and the degradation of H_2_O_2_ by catalases [7]. Thus, peroxisomes are both sources and sinks of ROS and account for a great part of oxygen consumption and ROS production (up to 35%) in metabolically active tissues such as the liver [4, 8]. Peroxisomes are much more dynamic organelles than anticipated, and the impact of the dynamic regulation of their size, abundance, morphology, and function in response to environmental stimuli or during development has only recently been addressed [9–11]. Given the dynamic nature of peroxisomes and their central role in fatty acid oxidation, an efficient quality control of this organelle is required to avoid oxidative damage and premature aging [3, 5, 12].

**Figure 1:**
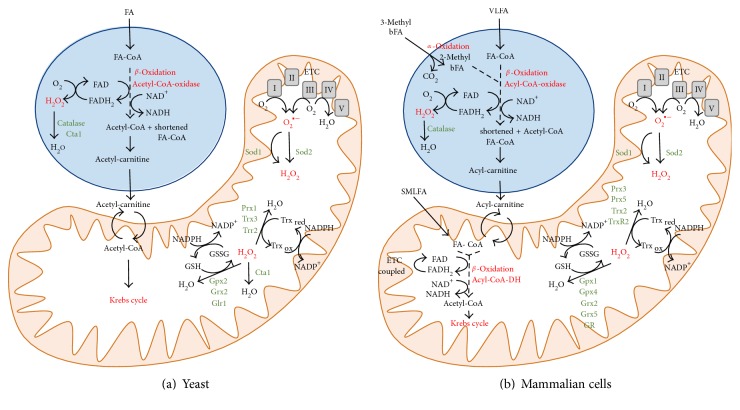
Pro- and antioxidant functions of peroxisomes and mitochondria in yeast and mammalian cells. Peroxisomes play important roles in the oxidative degradation of fatty acids. The peroxisomal fatty acid oxidation pathways are schematically depicted for budding yeast (a) and mammalian cells (b). Mitochondrial pro- and antioxidant functions and their interconnection with peroxisomes are summarized. ROS-generating and ROS-scavenging functions are highlighted for both organelles. FA = fatty acid; bFA = branched fatty acid; VLFA = very long fatty acid; SMLFA = small, medium, and long fatty acids; FA-CoA = fatty acyl coenzyme A; ETC = electron transport chain; Gpx = glutathione peroxidase; Grx = glutaredoxin; GR and Glr = glutathione reductase; Sod = superoxide dismutase; GSH = reduced glutathione; GSSG = oxidized glutathione; Prx = peroxiredoxin; Trx = thioredoxin; Trr and TrxR = thioredoxin reductase; DH = dehydrogenase; I to V = mitochondrial respiratory complexes I–V.

The oxidative degradation of fatty acids takes place in peroxisomes and mitochondria (Figure 1()). However, the compartmentalization of fatty acid oxidation pathways between these two organelles is variable in different organisms. Budding yeast, for example, degrades fatty acids exclusively in peroxisomes via *β*-oxidation, which implies the removal of two carbons from the fatty acid carboxyl terminus [13]. The first step in this process is catalyzed by acyl-CoA oxidases, which reduce O_2_ to H_2_O_2_. The end product of *β*-oxidation in yeast, acetyl-CoA, is transformed to acetyl-carnitine by peroxisomal carnitine acetyl-CoA transferases and shuttled into mitochondria to fuel the Krebs cycle and respiration. In human cells, mitochondria and peroxisomes cooperate in different routes of fatty acid oxidation [14]. Here, fatty acids with shorter chain lengths (C_6_-C_20_) are oxidized by *β*-oxidation in mitochondria, where the first step is catalyzed by acyl-CoA dehydrogenases coupled to the mitochondrial electron transport and not to H_2_O_2_ production. However, fatty acids with longer chain lengths (>C_20_) or dicarboxylic fatty acids have to be degraded by the peroxisomal *β*-oxidation pathway. Additionally, 3-methyl-branched fatty acids are exclusively metabolized in human peroxisomes by the process of *α*-oxidation, which represents an additional source of the ROS H_2_O_2_ originating from these organelles. In the human case, *β*-oxidation is not necessarily carried out to completion in peroxisomes; hence, the final product is a shortened fatty acyl-CoA, which is shuttled to mitochondria in the form of the corresponding fatty acyl carnitine conjugate. It is worth noting that ROS production by mammalian peroxisomes is not limited to the fatty acyl-CoA oxidases of the *β*-oxidation pathway. Additionally, oxidases participating in glyoxylate metabolism, amino acid catabolism, or the oxidation of polyamines are potential ROS sources of the peroxisome [15].

Peroxisomes contain several antioxidant systems, which are important for ROS homeostasis of this highly oxidative organelle (Figure 1). Since the *β*-oxidation pathway directly produces H_2_O_2_, the detoxifying catalase activity is of central importance for the redox balance of the organelle [16, 17]. It has been shown in yeast that peroxisomal catalase is required also for the tolerance to externally added H_2_O_2_, which suggests that peroxisomes are generally important for cellular ROS detoxification [18]. An additional peroxisomal antioxidant activity has been described in yeast as the glutathione peroxidase Gpx1, whose activity is generally required for a proper peroxisomal biogenesis [19]. Also in mammalian cells, catalase is the predominant antioxidant activity of peroxisomes. However, several other ROS detoxifying are known in higher eukaryotes to contribute to peroxisomal redox balance, which include superoxide dismutases, peroxiredoxins, glutathione S-transferases, and epoxide hydrolases [5].

The importance of peroxisomes in the maintenance of a healthy redox balance has been derived from situations, which interfere with a normal peroxisomal function and biogenesis. Artificial stimulation of peroxisomal biogenesis by the long-term exposure to peroxisomal proliferators causes oxidative liver damage and ROS imbalances in general in rodents [20, 21], most likely by the uncompensated induction of ROS-generating peroxisomal enzymes [22]. However, stimulated peroxisomal proliferation seems to reduce ROS levels in murine neurons [23]. Moreover, the loss of peroxisomes increases the apoptotic damage in the cerebellum and neurodegeneration [24, 25], and a deficiency in human catalase activity increases the susceptibility to aging-related diseases such as diabetes, cancer, or atherosclerosis [26]. Moreover, catalase mutants show phenotypes of accelerated aging in the Caenorhabditis model [27]. In yeast, loss of catalase function modulates the lifespan depending on the metabolic activity of peroxisomes [18]. Thus, peroxisomal (together with mitochondrial) activity in maintaining the cellular redox state is generally important for cell survival and health [28, 29].

## 3. Pro- and Antioxidant Functions of Mitochondria

Mitochondria have been traditionally described as the cellular power-house as they are the main contributors of energy through oxidative phosphorylation and the biosynthesis of ATP. Structurally, they contain two biologically different membrane systems: the outer membrane containing the intermembrane space and the inner membrane containing the mitochondrial matrix. The different electron transport chain (ETC) protein complexes are embedded in the inner membrane, which is heavily folded into the cristae structure [30]. Apart from regulating the energy metabolism of the eukaryotic cell, mitochondria are involved in many other fundamental processes, such as the control of the cell cycle or the induction or prevention of cell death [31–33].

Generation of ATP by the mitochondrial oxidative phosphorylation is based on the reduction of molecular oxygen, which can produce toxic byproducts such as the superoxide radical (O_2_^_^) or hydrogen peroxide (H_2_O_2_). Only complex IV (cytochrome *c* oxidase) of the ETC is able to fully reduce molecular oxygen to water and therefore has an antioxidant function in the ETC, while mainly complex I (NADH ubiquinone oxidoreductase) and complex III (ubiquinol-cytochrome *c* oxidoreductase) contribute to superoxide radical formation due to incomplete O_2_ reduction [34]. These ROS can trigger the formation of even more harmful secondary ROS such as the hydroxyl radical (^.^OH), which are supposed to be responsible for the main oxidative damage derived from mitochondrial activity [35, 36]. Electron leakage from mitochondrial electron transport chains is now recognized as one of the most important intracellular sources of ROS including hydroxyl radicals [37]. Mitochondria possess their own antioxidant systems, which consist of various ROS-scavenging enzymes and low molecular antioxidants [38, 39]. Primary antioxidant enzymes are superoxide dismutases (SOD), which reduce superoxide radicals to hydrogen peroxide. Two types of SOD can be found in mammalian mitochondria: manganese superoxide dismutase (SOD2) in the mitochondrial matrix and copper/zinc superoxide dismutase (SOD1) in the intermembrane space [40–42]. Detoxification of mitochondrial H_2_O_2_ can be achieved by the activity of glutathione peroxidases (GPx) and glutaredoxins (GRx). At least two GPx isoforms have been identified in the mammalian mitochondria, GPx1 and GPx4 [43, 44], and two Grx isoenzymes are active in mitochondria, Grx2 and Grx5 [45]. During this reaction, glutathione is oxidized, which has to be reduced by mitochondrial glutathione reductase (GR) with the help of the mitochondrial NADPH pool. Additionally, hydrogen peroxide can be reduced by mitochondrial peroxiredoxins (Prx) coupled to the oxidation of thioredoxins (Trx), which are finally recycled at the expense of NADPH by Trx reductases (TrxR). In the mammalian mitochondria, at least the isoenzymes Prx3, Prx5, and Trx2 have been described [46–50]. Among the nonenzymatic antioxidants found in the mammalian mitochondria, the high NADPH pool, vitamin C, and coenzyme Q have been reported with important functions in ROS elimination [51–53]. As opposed to peroxisomes, significant catalase activity is absent from the mammalian mitochondria. Yeast mitochondria also contain a full set of antioxidant systems based on glutathione or thioredoxin with the enzymes Gpx2, Grx2, Glr1, Prx1, Trx3, and Trr2 [54]. Also in yeast, the Sod1 Cu/Zn- and the Sod2 manganese-superoxide dismutases are the primary ROS-reducing enzymes in mitochondria [55, 56]. Additionally, and different to the situation in mammalian cells, the peroxisomal catalase Cta1 can also be found inside the yeast mitochondria [57].

The free radical theory of aging first postulated that the accumulation of cellular damage caused by free radicals was a decisive driving force in the process of aging and in the determination of a lifespan [58]. A major prediction derived from this theory was that the supplementation with antioxidants or genetic manipulation of endogenous ROS-scavenging systems would have a beneficial effect on the lifespan of the organism. However, no conclusive results were obtained by studies of this type in general [59–64], which led to a revision of the original hypothesis in a form of the mitochondrial-free radical theory [65]. Here, mitochondrially generated free radicals were supposed to have a major effect on aging, and thus it was proposed that reinforcement of mitochondrial antioxidant systems had lifespan extension effects. On the contrary, mitochondrial damage or insufficient ROS-scavenging activity of mitochondria could lead to lifespan shortening. A large body of evidence has accumulated, which supports this hypothesis [66–68]. Artificial targeting of catalase to mitochondria extends the lifespan of mice [69]. Similarly, the mitochondria-targeted antioxidant SkQ1 has lifespan extension effects in several rodent models [70]. On the contrary, mouse models, with mutations in the mitochondrial DNA polymerase gamma, have a shorter lifespan and manifest several phenotypes of accelerated aging [71]. Furthermore, the accumulation of damage in the mitochondrial DNA (mtDNA) caused in these mouse models has been reported to favor apoptosis and age-related diseases [72] and can be reverted by mitochondria-targeted catalase [73]. Moreover, deficiencies in Mn-superoxide dismutase activity increases oxidative stress and promotes aging in Drosophila [74–76]. Yeast has very efficiently contributed to reveal physiologically relevant determinants of lifespan and health span in higher organisms [77, 78]. Also in this simple model, mitochondria have been identified with an important role in the establishment of lifespan [79]. Specifically, yeast *sod* mutants are known to critically interfere with longevity [80]. Having found that mitochondrial ROS production is a driving factor of aging, a growing number of studies now intend to reduce ROS production by the targeted delivery of antioxidants to the mitochondria [1]. These approaches include the use of mitochondria-directed redox agents to detoxify mitochondrial ROS [81] or modulators of mitochondrial electron transport and leakage to avoid mitochondrial ROS [82]. These strategies are beyond the scope of this review; hence, the interested reader might consult summaries made by excellent recent revisions [68, 83, 84].

It is important to note that ROS might not always trigger fatal oxidative damage within a cell. On the contrary, low ROS concentrations have important biological functions related to signaling and stress resistance [85–87]. It has been increasingly clear in the last years that oxidative stress is able to actually promote longevity, which has led to the theory of mitochondrial hormesis. Here, stimuli such as calorie restriction or exercise are supposed to trigger adaptations, which lead to the reinforcement of endogenous antioxidant systems, increased stress tolerance, and subsequently slow down the aging process [88, 89]. There are many experimental findings in different model systems including yeast, Caenorhabditis or Drosophila, which are in line with the mitohormesis hypothesis [90, 91]. Inhibition of respiration, for example, increases the lifespan of nematodes dependent on the production of mitochondrial ROS [92]. Calorie restriction and especially glucose restriction partially induce mitochondrial metabolism and prevent aging from yeast to flies [93, 94]. It has been demonstrated in Caenorhabditis that reduced sugar intake induces mitochondrial respiration and ROS production, which are necessary for lifespan extension, and this beneficial effect is reverted by antioxidants [95]. In yeast, several interventions including the inhibition of nutrient-sensing protein kinases or dietary restriction cause an extended lifespan [96]. These effects have been shown to depend on mitochondrial activity in general or specific mitochondrial ROS-scavenging activities [97–100]. Taken together, mitochondrial ROS balance is decisive for the modulation of longevity and we will continue to summarize how mitochondrial and peroxisomal activity might change during the process of aging and in age-related diseases.

## 4. Compromised Function of Peroxisomes and Mitochondria during Aging

The investigation of the detrimental function of peroxisomal dysfunction in the process of aging has traditionally lagged behind mitochondria, which were considered the major source of oxidative stress-related senescence. However, it is much more considered now that a misregulated peroxisomal ROS balance is important in the occurrence of age-related diseases [3, 101, 102]. Recently, it has been shown that a deficiency in peroxisomal ROS clearance actually affects mitochondrial function. Specifically, inhibition of peroxisomal catalase activity negatively modulates the redox balance in mouse mitochondria [103] and human fibroblast [104]. Moreover, a localized oxidative damage to peroxisomes leads to mitochondrial fragmentation and cell death [105, 106]. These results reflect the intimate relation of both organelles in the maintenance of the cellular redox balance and furthermore suggest that peroxisomal dysfunction can subsequently damage mitochondria. Therefore, peroxisomes can act at the forefront in the initiation of cellular oxidative damage and aging [3]. Accordingly, several reports support that peroxisomal function continuously declines during aging. In cultured human cells, it has been shown that catalase is increasingly excluded from peroxisomes after repeated cell passage. At the same time, old cells accumulate more dysfunctional peroxisomes which raise cellular ROS levels and ultimately might accelerate aging [107]. In line with these results is the finding that in Caenorhabditis the abundance of many peroxisomal proteins decays during aging. Importantly, this affects the Pex5 importer of peroxisomal matrix proteins [108]. Pex5 function has been found to be regulated by the redox state of the organelle [109, 110]. Thus, the decline of peroxisomal protein import by oxidative stress, including the import of antioxidants, might be a trigger for senescence. Additionally, it has been shown that yeast peroxisome proliferation is regulated by the redox state of the organelle [19]. Oxidative stress might inhibit yeast peroxisomal biogenesis via multiple targets, such as Pex5 or Pex11 [109, 111–113]. Finally, interference with peroxisomal fission increases the lifespan of yeast cells [114]. Taken together, maintaining functional peroxisomes emerges as an important determinant of the cellular lifespan. The relevant mechanisms of peroxisomal homeostasis will be discussed in the following section.

A large body of experiments has demonstrated that mitochondrial dysfunction is an important trigger for cellular senescence, which contributes to aging in addition to other prosenescence stimuli [115–118]. Although it is clear that dysfunctional mitochondria accumulate in senescent cells and that this is a major driving force for accelerated aging [119–121], it is less understood how mitochondria become dysfunctional during the aging process [122, 123]. Mitochondrial defects which accumulate in older cells range from an increased mitochondrial mass, a decrease of respiratory coupling, less efficient ATP production during respiration, loss of respiratory complex I function, and increased mitochondrial ROS production [117, 118].

Importantly, there is increasing evidence that misregulated ROS production is an important trigger for mitochondrial dysfunction in the context of several age-related diseases. Cardiomyopathy in the old age, for example, is accompanied by an impairment of oxidative phosphorylation in heart mitochondria [87, 124]. Consequently, the rate of ROS production increases with age especially in cardiac mitochondria [125]. Similarly, an inefficient mitochondrial energy metabolism has been linked to cardiovascular diseases [126, 127]. In order to explain this decline in mitochondrial function, several mechanisms have been suggested, for example, an impaired biogenesis of the organelle, increased mitochondrial uncoupling, or the accumulation of mt-DNA mutations [128–130]. Mitochondrial dysfunction has been broadly implied in age-related neurodegenerative diseases [131–133]. In the particular case of Alzheimer's disease, the accumulation of the toxic aggregates of the unprocessed *β*-amyloid peptide is known to inhibit the mitochondrial electron transport chain, cause ROS overproduction, and induce mitochondria-mediated cell death [134–138]. Additionally, the accumulation of mt-DNA aberrations and failures in the dynamic regulation of mitochondrial morphology by fission have been linked to Alzheimer's disease [139–141]. Parkinson's disease is another neurodegenerative disorder where mitochondrial dysfunction has been identified as a major driving force. Several genes have been associated with familial Parkinson, and most of these PARK loci are functionally related with mitochondria [142]. PARK1 for example encodes *α*-synuclein, which can inhibit mitochondrial fusion [143]. Mutations in PARK7 affect DJ-1 function causing defects of mitochondrial respiratory complex I, ROS overproduction, and a loss of mitochondrial membrane potential [144, 145]. PARK8 encodes the LRRK2 protein kinase, whose function is needed for proper oxidative phosphorylation activity and mitochondrial fission [146, 147]. PARK2 and PARK6 encode Parkin and PINK1 (PTEN-induced kinase 1), which are involved in the correct turnover and degradation of dysfunctional mitochondria by mitophagy [148, 149]. The mechanisms of autophagic removal of excess or malfunctioning mitochondria and peroxisomes will be described in the following section.

Mitochondria form dynamic networks, whose morphology changes in response to stress and nutritional stimuli and damage [150, 151]. Several independent experimental evidences exist showing that changes in mitochondrial dynamics are intimately linked to senescence and accelerated aging [152]. During aging, mitochondrial fission is reduced, which leads to mitochondrial elongation [153]. It has been shown that inhibition of the profission protein FIS1 or the E3 ubiquitin ligase MARCH5, which positively regulates mitochondrial fission, induces senescence via mitochondrial elongation [154, 155]. In yeast, the pharmacological repression of mitochondrial fission leads to a lifespan extension [154, 156]. These results indicate that mitochondrial fission is needed for a normal cellular lifespan. This is in agreement with the finding that hyperelongated mitochondria have lower membrane potentials and excessively produce ROS [153, 154]. Additionally, the reinforcement of mitochondrial fission by the overexpression of FIS1 can reduce age-related phenotypes [153]. Importantly, mitochondrial fission is needed for efficient clearance of defective mitochondria via mitophagy, which could additionally explain the antiaging effect of the fission process [157, 158]. In a reverse manner, oxidative stress alters mitochondrial morphology via the modulation of several proteins involved in mitochondrial dynamics and this phenomenon has been linked to cancer progression [159–161]. Exogenous ROS efficiently inhibit mitofusins (Mfn1 and Mfn2) in human fibroblasts and muscle myoblasts inducing mitochondrial fission and membrane depolarization [162, 163]. In yeast, oxidative stress promotes mitochondrial fragmentation via the stimulation of the assembly of the mitochondrial fission machinery composed of Mdv1, the Dnm1 GTPase, and the Fis1 mitochondrial fission proteins [164].

Another important question is whether the asymmetric inheritance of mitochondria of different qualities or activities modulates the aging process. This phenomenon has been extensively studied in the yeast model of replicative aging [165]. During cell divisions, proaging components such as damaged or dysfunctional mitochondria are retained in the older mother cell and not transmitted to the young daughter cell [166]. Specific mitochondrial tether proteins such as Mmr1 have been identified, which actively retain highly oxidative mitochondria in the mother cell. Consequently, interference with this asymmetric mitochondrial distribution by deletion of Mmr1 shortens the yeast lifespan [167]. Additional anchor proteins such as Mfb1 have been identified now, which guarantee the remaining of highly functional mitochondria in the mother cell [168]. Interestingly, the unequal distribution of mitochondria from the mother to daughter cell is conditioned by mitochondrial dynamics since it is impaired in mitochondrial fusion mutants [169]. Taken together, the fusion/fission process of mitochondria (and peroxisomes) is important for the maintenance of a healthy redox balance and lifespan. The quality control of both organelles via autophagic mechanisms is of key importance to maintain both mitochondria and peroxisomes functional.

## 5. Quality Control of Peroxisomes and Mitochondria by Autophagy and the Impact on Health

**Figure 2:**
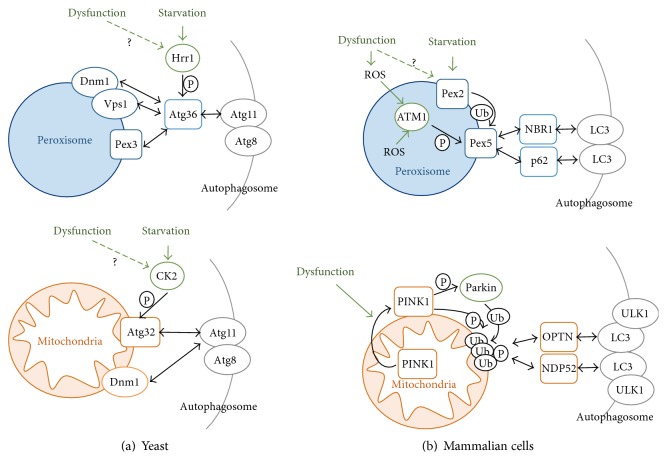
Mechanisms of autophagic removal of peroxisomes and mitochondria. In the upper panel, pexophagy mechanisms are depicted for budding yeast (a) and mammalian cells (b). In yeast, pexophagy is induced by the Hrr1 kinase which phosphorylates the Atg36 adaptor. Atg36 contacts the Pex3 peroxisomal receptor, the fission machinery (Dnm1, Vps1), and the autophagosomal adaptor Atg11. In mammalian cells, a dysfunctional peroxisome and general ROS increase activate the ATM kinase, which phoshorylates the Pex5 receptor. Pex5 is additionally targeted by ubiquitination via the starvation-inducible Pex2. Modified Pex5 interacts with the autophagosomal adaptors NBR1 and p62. In the lower panel, mitophagy mechanisms are depicted for budding yeast (left) and mammalian cells (right). In yeast, starvation induces mitophagy via casein kinase 2 (CK2), which phosphorylates the Atg32 receptor. Modified Atg32 interacts with the Atg11 autophagosomal adaptor, which also contacts the mitochondrial fission machinery (Dnm1). In mammalian cells, mitochondrial dysfunction triggers the exposure of PINK1 at the organelle surface. PINK1 phosphorylates and activates the Parkin ubiquitin ligase, which marks outer mitochondrial membrane proteins. PINK1 additionally phosphorylates polyubiquitin chains at mitochondria, which leads to recognition by the autophagosomal adaptor proteins Optineurin (OPTN) and NDP52. P = phosphorylation; Ub = ubiquitination.

The number, activity, and quality of peroxisomes are dynamically adapted and controlled in the cell [170]. An important mechanism to eliminate dysfunctional or superfluous peroxisomes consists in a specific autophagy termed pexophagy [171]. The mechanisms which assure the engulfment of specific peroxisomes in autophagosomes and their subsequent degradation in vacuoles (fungi) or lysosomes (mammals) have been especially elucidated in the yeast model [172]. Here, pexophagy has been intensively studied in response to nutritional changes from respiratory to fermentative growth conditions, which implies the proteolytic removal of excess peroxisomes [172]. The key step in the initiation of pexophagy is the formation of a preautophagosomal structure at the surface of the organelle [173–176] (Figure 2). Several Atg proteins are coordinated in this process termed cytoplasm-to-vacuole targeting; however, the Atg11 cytosolic adaptor protein is the physical link of the organelle to be degraded and the autophagosome in the case of both peroxisomes and mitochondria [175, 176]. Atg11 contacts the peroxisome via a specific adaptor protein at the peroxisomal surface. In budding yeast, the specific adaptor for pexophagy is Atg36, which directly interacts with the peroxisomal Pex3 protein and the core phagosomal components Atg11 and Atg8 [177, 178]. One way to induce pexophagy is through the phosphorylation of Atg36 by the Hrr25 casein kinase homolog [179]. Hrr25 phosphorylates a specific serine residue (S97) in the Atg36 adaptor, which triggers its interaction with Atg11 but not with Pex3 [179]. Therefore, phosphorylation of Atg36 by Hrr25 is a switch in the initiation of pexophagy by allowing the interaction of the peroxisome with the central autophagosomal machinery [179]. Peroxisomes have their own fission system to allow an autonomous proliferation of the organelle. In yeast, the dynamin-related small GTPases Dnm1 and Vps1 together with the fission protein Fis1 are required for peroxisomal fission [180–182]. It has been shown recently that peroxisomal fission is required for pexophagy [183]. Moreover, the Atg11 adaptor protein interacts directly with Dnm1 and Vps1 [183]. Taken together, the Atg11 mediated recruitment of the preautophagosomal structure at strategic sites of the peroxisome via Pex3, and the fission machinery seems to be the initial step for marking peroxisomes for proteolytic degradation. It is less understood whether and how pexophagy serves to eliminate dysfunctional peroxisomes. However, it has been shown in yeast that artificially created catalase aggregates are cleared in a process that depends on peroxisomal fission and pexophagy [184]. Additionally, peroxisomal protein import defects have been shown to increase the rate of pexophagy [185]. These results suggest that the autophagic removal of damaged peroxisomes with increased ROS production could be an important mechanism to maintain a correct redox balance in the cell [172]. Consistent with this idea is the finding that peroxisomal proliferation seems to be regulated by the redox state of the organelle [19] and that abiotic stress such as salt stress induces the number of peroxisomes via Dnm1 and Vps1 [186].

The mechanisms of pexophagy in mammalian cells are only beginning to be unraveled [187, 188]. Here, selective autophagy of peroxisomes relies on cytosolic adaptor proteins such as p62 or NBR1, which recognize ubiquitinated cargo proteins and direct them to the autophagosome [189] (Figure 2). Pex5 is the peroxisomal receptor which upon ubiquitination triggers the process of pexophagy [190, 191]. This process can be stimulated upon external oxidative stress or by the pharmacological induction of peroxisomal fatty acid oxidation and involves the ATM kinase [191, 192]. Activated ATM inhibits signaling through the mammalian TOR pathway and marks Pex5 for pexophagy by phosphorylation [193]. Subsequently, Pex5 is ubiquitinated, and p62-dependent autophagy is initiated [191]. An E3 ubiquitin ligase responsible for Pex5 ubiquitination has been recently found with Pex2 at least upon nutrient starvation conditions [194]. Taken together, these findings suggest that an excess of ROS produced at peroxisomes triggers the process of specific pexophagy to maintain a healthy redox balance in mammalian cells.

The removal of excess or damaged mitochondria by selective autophagy or mitophagy is an essential cellular homeostasis mechanism. In yeast, this process has been heavily studied upon nutrient starvation conditions, for example, upon depletion of a N-source or in the stationary growth phase. These approaches have revealed many molecular insights of mitophagy; however, it remains unclear whether mitophagy in yeast actually degrades dysfunctional organelles [195]. Of central importance is the mitochondrial mitophagy receptor protein Atg32, which is localized at the outer mitochondrial membrane [196, 197]. Upon induction of mitophagy by starvation, Atg32 is phosphorylated by casein kinase 2 (CK2) at a specific serine (S114) residue [198]. Phosphorylation of Atg32 triggers its interaction with the cytosolic Atg11 adaptor protein. Therefore, this step is considered the initial signal in the targeting of mitochondrial to the preautophagosomal structure. Interestingly, there are at least two additional kinases which have been reported to be necessary for Atg32 phosphorylation, the Hog1 and Slt2 MAP kinases [199, 200]. Although both kinases cannot directly phosphorylate, the Atg32 receptor, the involvement of both kinases in stress signaling, could indicate a possible regulation of mitophagy by cellular stress stimuli additionally to nutrient shortage. Although still a matter of debate, there are experimental indications which suggest that mitophagy in yeast depends on mitochondrial fission. Generally, the mitochondrial fission proteins Fis1, Dnm1, Mdv1, and Caf4 seem to require for efficient mitophagy [157, 158, 201]. Specifically, during mitophagy, Atg11 directly targets the mitochondrial Dnm1 fission protein [158]. This suggests that mitophagy takes place close to the mitochondrial fission apparatus which would be necessary to separate the mitophagic cargo from the rest of the mitochondrial network [157]. Additional insights into the specific origins of mitophagy have been recently revealed by the finding that specific contact sites between the ER and mitochondria, the so called ERMES (ER mitochondria encounter structure), are necessary for mitophagy [202]. Thus, the initiation of mitophagy might require a close contact between the mitochondrial fission apparatus and the ER at strategic sites. It is less understood whether mitophagy in yeast is necessary to avoid cellular damage by dysfunctional mitochondria or whether it is activated upon oxidative stress. However, it has been reported that autophagic removal of excess mitochondria upon nutrient starvation is required to decrease intracellular ROS levels [203].

In mammalian cells, mitophagy has been intensively studied upon conditions which damage mitochondria by counteracting and eliminating their normal membrane potential [204] (Figure 2). In fact, loss of the mitochondrial membrane potential is one of the main triggers of mitophagy in mammalian models. Modulation of the mitochondrial shape is another important step in mitophagy, and inhibition of mitochondrial fission impairs the sequestration of damaged mitochondria by autophagosomes [205, 206]. This suggests that fission of the organelle, which is often induced by cellular stress, is an important mechanism to segregate and eliminate damaged mitochondria from the otherwise healthy network. Failure in the execution of mitophagy surveillance pathways clearly impairs the cellular homeostasis. This has been demonstrated for the key regulators of mitophagy, PINK1 (PTEN-induced kinase), and Parkin, whose mutation can cause Parkinson's disease [207, 208]. PINK1 is a mitochondrial serine/threonine kinase encoded by the *PARK6* locus [208], while Parkin is a cytosolic E3 ubiquitin ligase encoded by *PARK2* [209]. Both proteins function in the same mitophagic pathway with PINK1 acting upstream of Parkin [210, 211]. Upon normal cell homeostasis, PINK1 is imported into mitochondria dependent on the mitochondrial membrane potential. In healthy mitochondria, it is cleaved by PARL at the inner mitochondrial membrane, thereby restricting its activity [212–214]. Loss of the mitochondrial membrane potential inhibits PINK1 import and provokes its accumulation at the outer mitochondrial membrane, where it recruits Parkin [215–217]. This is considered the key step to mark dysfunctional mitochondria for selective degradation. Once exposed at the mitochondrial surface, PINK1 forms larger aggregates, which stimulate PINK1 activity by autophosphorylation [218, 219]. Subsequently, the mitophagic pathway is initiated by PINK1 phosphorylation of both Parkin and ubiquitin in a positive feedback regulation. The phosphorylation of Parkin by PINK1 is necessary for its mitochondrial targeting and for its E3 ubiquitin ligase activity [215, 216, 220–223]. Furthermore, PINK1 phosphorylates ubiquitin, and phosphorylated ubiquitin seems to be the true targeting signal for Parkin [224–226]. Indeed, it has been recently revealed that Parkin accumulation at damaged mitochondria requires phosphorylated ubiquitin chains at the mitochondrial surface [227–229]. Ubiquitination of outer mitochondrial membrane proteins, most importantly of the mitofusins Mfn1/2, finally triggers the physical segregation of the damaged mitochondrial parts and degradation via autophagosomes [230–232]. The nature of the autophagy receptors which would recognize ubiquitinated damaged mitochondria has been controversial. However, a recent study discards the pexophagy receptors p62 or NBR1 and specifically implies Optineurin and NDP52 in PINK1/Parkin-mediated mitophagy [233]. Thus, phospho-ubiquitin at the surface of damaged mitochondria is the signal to recruit the NDP52 and Optineurin receptors, which in turn contact with components of the autophagy pathway such as ULK1 or LC3 to engage in the autophagic degradation of the dysfunctional mitochondria [234, 235].

It is important to note that mammalian cells apparently use different mitophagy pathways upon different stimuli. It has been demonstrated in mouse and human cells that oxidative stress is a sensitive mitophagy trigger, which reinforces the idea that the autophagic removal of damaged mitochondria is a physiologically relevant mechanism of cell homeostasis [236]. Apart from the PINK1/Parkin system, which responds to a loss of mitochondrial membrane potential, the Bcl2-L-13 protein (yeast Atg32 ortholog), the NIX/BNIP3L, and the FUNDC1 mitochondrial outer membrane proteins have been involved in different types of mitophagy. Bcl2-L-13 seems to mediate mitophagy and mitochondrial fragmentation upon ETC damage [237], NIX/BNIP3L is involved in mitochondrial clearance during erythrocyte differentiation [238, 239], while FUNDC1 responds to hypoxic conditions [240]. It remains unclear to what degree these different mitophagy receptors act separately in the process of mitochondrial homeostasis.

## 6. Physiological and Physical Interaction of Peroxisomes and Mitochondria

In mammalian cells, peroxisomes and mitochondria functionally cooperate in the oxidative degradation of fatty acids by *β*-oxidation (see Figure 1). Both organelles have different but partially overlapping substrate specificities with regard to the chemical structure of the fatty acid to be metabolized. Therefore, peroxisomes and mitochondria cooperate in the homeostasis of lipids. The details of this metabolic cooperation have been summarized in excellent recent reviews [241, 242]. It is important to note that both organelles share metabolic pathways apart from *β*-oxidation, for example in the detoxification of glyoxylate or in the degradation of special fatty acids via *α*-oxidation [241, 243]. Also, it can be assumed that extensive peroxisomal *α*-oxidation depends on the ATP supply from the mitochondrial respiratory chain. More importantly, it has been recently shown that there is an intimate crosstalk between peroxisomes and mitochondria in the homeostasis of intracellular ROS beyond the fact that both organelles are important sources and sinks for ROS as detailed above. It had been known that weakening the peroxisomal catalase activity either by pharmacological treatment or caused by the decreased protein import capacity triggered elevated oxidative stress and reduced enzyme activity at mitochondria [104, 107, 244]. The use of peroxisomally directed inducers of oxidative stress has then revealed that mitochondria indeed are downstream of peroxisomally generated ROS [101], which is manifested by mitochondrial fragmentation and cell death [105, 106]. It has been suggested that mitochondrial oxidative damage is caused by lipid peroxidation originated in peroxisomes arguing against a simple diffusion of peroxisomal ROS to mitochondria. The mechanisms of the peroxisome-mitochondria ROS interplay are currently unknown; however, interorganelle contact sites could explain this much more intimate relation of peroxisomes and mitochondria.

**Figure 3:**
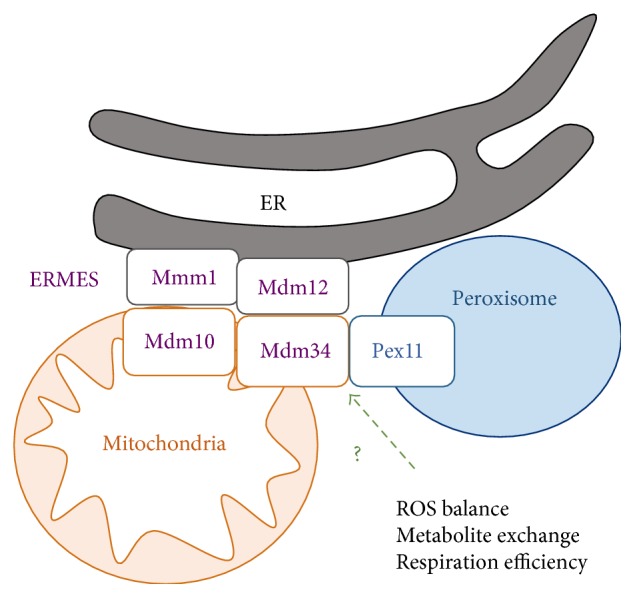
Overview of the yeast peroxisome-mitochondria contact site. The ERMES (endoplasmic reticulum mitochondria encounter structure) tethers mitochondria to the ER, but additionally establishes a contact between mitochondria and peroxisomes through the Mdm34-Pex11 interaction. The mitochondria-peroxisome tether might have dynamic functions in the regulation of ROS homeostasis, metabolite exchange between the two organelles, or the modulation of respiratory efficiency.

In the yeast model, the functional and physical interactions of peroxisomes with mitochondria have been studied intensively in recent years. As outlined above, fatty acid *β*-oxidation occurs exclusively in peroxisomes of budding yeast. Therefore, acetyl-CoA from peroxisomes has to be channeled into mitochondria via the carnitine shuttle and thus a direct contact of both organelles should favor this exchange of metabolites. Indeed, it has been shown that yeast peroxisomes preferentially localize close to specific mitochondrial sites which also contain contacts to the ER [245]. Recently, a possible physical tether between peroxisomes and mitochondria has been suggested by the interaction of the Pex11 protein involved in the biogenesis of the organelle with the mitochondrial Mdm34 protein [246] (Figure 3). Since Mdm34 is one of the structural components of the mitochondria-ER tether ERMES [247], this finding opens up the possibility that at least in yeast peroxisomes and mitochondria are physically connected at specific contact sites with the ER [248]. Recent evidence suggests that peroxisome-mitochondria contact sites might also exist in mammalian cells [249]. Since peroxisomes and mitochondria share components of their fission machineries and the fission of both organelles is important for their autophagic turnover, it is possible that this three-way contact is structurally important for the regulated turnover and therefore the ROS homeostasis of both organelles. Additionally, the mitochondria peroxisome interaction might be regulated dynamically to modulate ROS homeostasis, the interchange of metabolites or the respiratory efficiency. In this respect, it has been recently shown that the number of peroxisomes attached to the mitochondrial network increases upon cellular stress such as high salinity, which is known to also increase cellular ROS levels [186].

## 7. Conclusions

We know that peroxisomes and mitochondria are the crucial organelles in the homeostasis of eukaryotic ROS levels. Peroxisomes have traditionally lagged behind the importance of mitochondria in the production and detoxification of intracellular oxidative stress; however, this difference has largely disappeared in the past years. Current investigation is now deciphering the functional interplay of both organelles in the propagation and avoidance of oxidative damage in the cell. Peroxisomal and mitochondrial oxidative stress are intimately linked. Moreover, the dynamic fission of both organelles shares structural components, and the turnover of damaged peroxisomes and mitochondria as a mechanism to reduce intracellular oxidative stress will still be an important field of investigation. From a structural viewpoint, we are only beginning to understand how molecular tethers between peroxisomes and mitochondria, most likely via the ER, can contribute to interorganelle communication and coordinated quality control of both organelles. Therefore, investigation of the peroxisome-mitochondria connection and its regulation by stress will continue to contribute essential knowledge of how the redox homeostasis is maintained in young, environmentally challenged and aged eukaryotic cells.
